# Hepatitis C Virus Infection among Injection Drug Users with and without Human Immunodeficiency Virus Co-Infection

**DOI:** 10.1371/journal.pone.0094791

**Published:** 2014-04-10

**Authors:** Meng-Hsuan Hsieh, Jih-Jin Tsai, Ming-Yen Hsieh, Chung-Feng Huang, Ming-Lun Yeh, Jeng-Fu Yang, Ko Chang, Wei-Ru Lin, Chun-Yu Lin, Tun-Chieh Chen, Jee-Fu Huang, Chia-Yen Dai, Ming-Lung Yu, Wan-Long Chuang

**Affiliations:** 1 Department of Preventive Medicine, Kaohsiung Medical University Hospital, Kaohsiung City, Taiwan; 2 Faculty of Medicine, School of Medicine, Kaohsiung Medical University, Kaohsiung City, Taiwan; 3 Division of Infectious Diseases, Department of Internal Medicine, Kaohsiung Medical University Hospital, Kaohsiung City, Taiwan; 4 Tropical Medicine Center, Kaohsiung Medical University Hospital, Kaohsiung Medical University, Kaohsiung City, Taiwan; 5 Department of Internal Medicine, School of Medicine, College of Medicine, Kaohsiung Medical University, Kaohsiung City,Taiwan; 6 Department of Internal Medicine, Kaohsiung Municipal Ta-Tung Hospital, Kaohsiung Medical University, Kaohsiung City, Taiwan; 7 Department of Occupational Medicine, Kaohsiung Municipal Ta-Tung Hospital, Kaohsiung City, Taiwan; 8 Hepatitis Center and Hepatobiliary Division, Department of Internal Medicine, Kaohsiung Medical University Hospital, Kaohsiung City, Taiwan; 9 Department of Internal Medicine, Kaohsiung Municipal Hsiao-Kang Hospital, Kaohsiung City, Taiwan; 10 Graduate Institute of Clinical Medicine, College of Medicine, Kaohsiung Medical University, Kaohsiung City, Taiwan; University of Pisa, Italy

## Abstract

The aim of this study is to explore the prevalence of hepatitis C virus (HCV) infection among injection drug users (IDUs) with and without human immunodeficiency virus (HIV) infection in southern Taiwan. For 562 IDUs (265 anti-HIV negative, 297 anti-HIV positive), we analyzed liver function, anti-HIV antibody, anti-HCV antibody, HCV viral loads, and hepatitis B surface antigen (HBsAg). HIV RNA viral loads and CD4 cell count for anti-HIV-seropositive IDUs and the HCV genotype for HCV RNA-seropositive IDUs were measured. The seroprevalence rates of anti-HIV, anti-HCV, and HBsAg were 52.8%, 91.3%, and 15.3%, respectively. All the anti-HIV-seropositive IDUs were positive for HIV RNA. Anti-HCV seropositivity was the most important factor associated with HIV infection (odds ratio [OR], 25.06; 95% confidence intervals [CI], 8.97–74.9), followed by male gender (OR, 6.12; 95% CI, 4.05–9.39) and HBsAg seropositivity (OR, 1.90; 95% CI, 1.11–3.34). Among IDUs positive for anti-HCV, 80.7% had detectable HCV RNA. HCV viremia after HCV exposure was strongly related to HIV infection (OR, 6.262; 95% CI, 1.515–18.28), but negatively correlated to HBsAg seropositivity (OR, 0.161; 95% CI, 0.082–0.317). HCV genotype 6 was the most prevalent genotype among all IDUs (41.0%), followed by genotypes 1 (32.3%), 3 (12.8%), and 2 (5.6%). In conclusion, about half IDUs were infected with HIV and >90% with HCV infection. Male and seropositivity for HBsAg and anti-HCV were factors related to HIV infection among our IDUs. HIV was positively correlated, whereas hepatitis B co-infection was negatively correlated with HCV viremia among IDUs with HCV exposure. Different HCV molecular epidemiology was noted among IDUs.

## Introduction

Hepatitis C virus (HCV) and human immunodeficiency virus (HIV) are found throughout the world, with estimated infection prevalences of 185 million [1] and 34 million [2], respectively. HCV and HIV share common routes of transmission, including injection drug use, sexual contact, and mother-to-child transmission during pregnancy or birth [3]. Therefore, injection drug users (IDUs) are at high risk of co-infection with HCV and HIV. In Taiwan, injection drug use has been the main route of transmission of HIV since 2005 [4]. Similar to the United States and Europe, where 33% of HIV-infected persons are co-infected with HCV [5]–[7], the prevalence of HCV co-infection among IDUs with HIV infection is not uncommon and has been increasing gradually in Taiwan [8], [9].

Previous studies showed that HIV infection exacerbates the natural history of HCV infection [10]–[13]. HCV patients co-infected with HIV are less likely to clear HCV viremia following acute infection, have higher HCV RNA loads, and experience more rapid progression of HCV-related liver disease than those without HIV co-infection. In addition, a longer duration of injection drug use increases the risk of HCV and HIV co-infection [14]. Hepatitis B virus (HBV) co-infection has been associated with spontaneous HCV seroclearance in the general population and end-stage renal disease patients [15], [16].

Both of HBV and HCV infection are epidemic in Taiwan, with a seroprevalence rate of 15.1% for HBV surface antigen (HBsAg) and 8.6% for antibodies to HCV (anti-HCV), respectively, in southern Taiwan [17]. In this study, therefore, we aimed to investigate the prevalence of HCV infection among IDUs with and without HIV infection and to explore the impact of HIV and HBV co-infections on HCV viremia among IDUs with HCV exposure.

## Patients and Methods

### Subjects

In Taiwan, prisoners who are IDUs gathered in certain appointed jails, and we recruited the IDU prisons from one such jail in southern Taiwan. Those selected for inclusion were IDUs who received a health checkup, were willing to enter our study, and were at least 18 years old. The exclusion criteria were a history of renal failure or severe heart failure. A total of 562 IDUs, 401 (71.4%) male and 161 (28.6%) female (mean age, 36.1 years), were recruited between March 2008 and June 2010. The study was approved by the ethics committee of Kaohsiung Medical University Hospital. Signed informed consent forms were obtained from all participants.

### Laboratory data

The following laboratory tests were performed on all subjects: aspartate aminotransferase (AST) and alanine aminotransferase (ALT) levels, HBsAg, anti-HCV antibody, HCV RNA, and anti-HIV antibody. HCV genotype was determined for subjects with detectable HCV RNA. HIV RNA viral load and CD4 cell count were determined for subjects seropositive for anti-HIV.

The aminotransferase levels were calculated as AST ratio, defined as the fold-change of the upper limit of normal AST range, and ALT ratio, defined as the fold-change of the upper limit of normal ALT range. HBsAg was determined by enzyme immunoassay (AUSAB EIA, Abbott Laboratories, North Chicago, IL, USA). Anti-HCV antibodies were detected with a commercially available third-generation enzyme-linked immunosorbent assay kit (Abbott Laboratories). HCV RNA viral loads were quantified by means of a real-time polymerase chain reaction assay (RealTime HCV; Abbott Molecular, Des Plaines IL, USA; detection limit: 12 IU/mL) [18]. Anti-HIV antibody assays (SERODIA-HIV, Fujirebio, Tokyo, Japan) were performed according to the manufacturer's instructions. HIV RNA viral loads were quantified by means of reverse-transcription polymerase chain reaction (Roche Amplicor, ver. 1.5, Roche Diagnostics, Branchburg, NJ, USA). CD4 counts were determined by flow cytometry (FACFlow, BD FACSCalibur, Becton Dickinson, San Jose, CA, USA). HCV genotype was determined by using a VERSANT HCV Genotype 2.0 Assay (Siemens Healthcare Diagnostics Inc., Tarrytown, NY, USA), according to the manufacturer's strict instructions.

### Statistical analysis

Student's *t*-test, χ^2^ test, Mann–Whitney *U*-test, and one-way analysis of variance were used to analyze and compare the data. Multivariate logistic regression analysis was used to evaluate the factors, such as gender, age, HBsAg status and anti-HCV, associated with HIV infection among IDUs, and factors, such as gender, age, HBsAg, and anti-HIV, associated with HCV infection among IDUs and HCV RNA viremia among IDUs with HCV exposure. All the tests were two-sided, and the significance levels were set at α = 0.05. Statistical analyses were performed with the SPSS statistical package (19th ed., SPSS Inc., Chicago, IL, USA).

## Results

The characteristics of the 562 IDUs enrolled in the current study are listed in Table 1. Of these, 297 (52.8%) were anti-HIV positive. All anti-HIV-seropositive subjects were seropositive for HIV RNA. The HBsAg and anti-HCV positivity prevalence rates were 15.3% and 91.3%, respectively.

**Table 1:** Characteristics and virological features of 562 IDUs with and without HIV infection.

	All	Anti-HIV(−)	Anti-HIV(+)	*p*
Total, no. (%)	562	265 (47.2)	297 (52.8)	
Age, years, mean ±SD	36.1±7.5	35.6±7.5	36.4±7.4	0.49
Gender				
Male, no. (%)	401 (71.4)	147 (55.5)	254 (85.5)	**<0.001**
Female, no. (%)	161 (28.6)	118 (44.5)	43 (14.5)	
AST ratio, U/L, mean ±SD^a^	0.97±0.70	0.86±0.59	1.06±0.76	**0.005**
ALT ratio, U/L, mean ±SD^a^	1.14±1.16	0.98±1.05	1.29±1.22	**<0.001** b
HBsAg status				
Negative, no. (%)	476 (84.7)	235 (88.7)	241 (81.1)	**0.013**
Positive, no. (%)	86 (15.3)	30 (11.3)	56 (18.9)	
Anti-HCV antibody status				
Negative, no. (%)	49 (8.7)	45 (17.0)	4 (1.3)	**<0.001**
Positive, no. (%)	513 (91.3)	220 (83.0)	293 (98.7)	
HCV RNA viral load, log (IU/mL), mean ±SD^c^		5.00±1.20	5.24±1.11	0.32
HIV RNA viral load, log (IU/mL), mean ±SD^d^		-	3.15±0.90	
CD4 cell count, cells/µL, mean ±SD			444.4±180.5	
>500 cells/µL, no. (%)			94 (31.6)	
350–500 cells/µL, no. (%)			100 (33.7)	
<350 cells/µL, no. (%)			103 (34.7)	

IDU, injection drug users; SD, standard deviation; AST, aspartate aminotransferase; ALT, alanine aminotransferase; HBsAg, hepatitis B surface antigen; HCV, hepatitis C virus

a Ratio relative to upper normal limit, the unit of AST and ALT is IU/L.

b Mann–Whitney *U*-test.

c For 173 HCV RNA detectable IDUs without HIV infection and 241 HCV RNA undetectable IDUs with HIV infection.

d All anti-HIV-positive subjects were seropositive for HIV RNA.

### Factors associated with HIV infection among IDUs

According to univariate analysis (Table 1), age was not different between anti-HIV-negative and -positive IDUs. Anti-HIV-positive IDUs had a significantly higher proportion of male gender (85.5% vs. 55.5%, *p*<0.001), anti-HCV seropositivity (98.7% vs. 83.0%, *p*<0.001), and HBsAg seropositivity (18.9%, vs. 11.3%, *p* = 0.013). The average AST and ALT ratios were significantly higher in IDUs with HIV infection than in those without (*p* = 0.005 and *p*<0.001, respectively). However, the HCV RNA viral loads among subjects with detectable HCV RNA did not differ between anti-HIV-seropositive and -seronegative IDUs.

Because both gender and anti-HCV were strongly associated with HIV infection, we performed a trend test to explore the interactive relationships among gender, anti-HCV, and HIV infection. The prevalence of HIV infection was 0% (0/5), 9.1% (4/44), 27.6% (43/156), and 70.0% (250/357), respectively, for anti-HCV-seronegative female patients, anti-HCV-seronegative male patients, anti-HCV-seropositive female patients, and anti-HCV-seropositive male patients (*p* for trend <0.001, Supplement Data, Figure S1). Multivariate analysis revealed that anti-HCV seropositivity was the most important factor associated with HIV infection (odds ratio [OR] 25.06; 95% confidence intervals [CI], 8.97–74.9), followed by male gender (OR, 6.12; 95% CI, 4.05–9.39), and HBsAg seropositivity (OR, 1.90; 95% CI, 1.11–3.34; Table 2).

**Table 2:** Multivariate analysis of factors associated with HIV infection status among IDUs.

Factor	Comparison	Odds ratio	95% confidence interval	*p* value
HBsAg status	negative = 0, positive = 1	1.90	1.11–3.34	**0.022**
Gender	female = 0, male = 1	6.12	4.05–9.39	**<0.001**
Anti-HCV antibody status	negative = 0, positive = 1	25.06	8.97–74.9	**<0.001**

HIV, human immunodeficiency virus; HBsAg, hepatitis B surface antigen; HCV, hepatitis C virus.

Factors used for logistic analysis included gender, age, hepatitis B surface antigen (HBsAg) status and anti-HCV antibody status.

### Factors associated with HCV exposure and HCV viremia among IDUs

We analyzed the factors associated with HCV exposure (anti-HCV seropositivity) among all IDUs (Table 3). In contrast to HIV infection, anti-HCV-seropositive IDUs had a significantly higher proportion of females (30.4% vs. 10.2%, *p* = 0.003), as well as significantly higher AST ratios (*p* = 0.01). The age, HBsAg status, and ALT ratio did not differ between IDUs with and without anti-HCV-seropositivity. Among anti-HIV-seropositive subjects, the HIV RNA viral loads and CD4 cell counts were also similar between IDUs with and without anti-HCV-seropositivity. Multivariate logistic analysis showed that HIV infection and female gender were significant factors associated with anti-HCV seropositivity with ORs (95% CI) of 25.03 (8.64–72.53) and 9.72 (3.67–25.74), respectively (Table 4).

**Table 3:** Factors associated with HCV exposure and persistent HCV viremia in IDUs.

	All IDUs			Anti-HCV(+) IDUs		
	Anti-HCV(−)	Anti-HCV(+)	*p* value	HCV RNA(−)	HCV RNA(+)	*p* value
Total, no. (%)	49 (8.7)	513 (91.3)		99 (19.3)	414 (80.7)	
Age, years, mean ±SD	34.1±6.7	36.3±7.5	0.42	36.0±7.8	36.5±7.3	0.18
Gender						
Male, no. (%)	44 (89.8)	357 (69.6)	**0.003**	61 (61.6)	296 (71.5)	0.055
Female, no. (%)	5 (10.2)	156 (30.4)		38 (38.4)	118 (28.5)	
HBsAg status						
Negative, no. (%)	41 (83.7)	435 (84.8)	0.84	66 (66.7)	369 (89.1)	**<0.001**
Positive, no. (%)	8 (16.3)	78 (15.2)		33 (33.3)	45 (10.9)	
AST ratio, U/L, mean ±SD^a^	0.63±0.37	0.99±0.71	**0.01**	0.92±0.64	1.06±0.76	0.08
ALT ratio, U/L, mean ±SD^a^	0.70±0.62	1.18±1.19	0.11	1.05±1.10	1.30±1.24	0.09
Anti-HIV antibody status						
Negative, no. (%)	45 (91.8)	220 (42.9)	**<0.001**	47 (47.5)	173 (41.8)	0.30
Positive, no. (%)	4 (8.2)	293 (57.1)		52 (52.5)	241 (58.2)	
HIV RNA viral load, log(IU/mL), mean ±SD^b^	3.39±0.85	3.14±0.90	0.94	2.91±1.13	3.19±0.84	0.09
CD4 cell count, mean ±SD						
>500 cells/µL, no. (%)	1 (25.0)	93 (31.7)	0.81	21 (40.4)	72 (29.9)	0.28
350–500 cells/µL, no. (%)	1 (25.0)	99 (33.8)		17 (32.7)	82 (34.0)	
<350 cells/µL, no. (%)	2 (50.0)	101 (34.5)		14 (26.9)	87 (36.1)	

SD, standard deviation; AST, aspartate aminotransferase; ALT, alanine aminotransferase; HBsAg, hepatitis B surface antigen; HCV, hepatitis C virus; HIV, human immunodeficiency virus.

a Ratio relative to upper normal limit, the unit of AST and ALT is IU/L.

b All anti-HIV-positive subjects were seropositive for HIV RNA.

**Table 4:** Multivariate analysis of factors associated with anti-HCV seropositivity for all IDUs and with HCV viremia among IDUs with HCV exposure.

Factor	Comparison	Odds ratio	95% confidence interval	*p* value
1Anti-HCV seropositivity among all IDUs				
Gender	male = 0, female = 1	9.72	3.67–25.74	**<0.001**
Anti-HIV status	negative = 0, positive = 1	25.03	8.64–72.53	**<0.001**
2HCV viremia among anti-HCV positive IDUs				
HBsAg status	negative = 0, positive = 1	0.161	0.082–0.317	**<0.001**
Anti-HIV status	negative = 0, positive = 1	6.262	1.515–18.28	**0.009**

HBsAg, hepatitis B surface antigen; HCV, hepatitis C virus; HIV, human immunodeficiency virus.

1 Factors used for logistic analysis included gender, age, HBsAg status, and anti-HIV status.

2 Factors used for logistic analysis included gender, age, HBsAg status, and anti-HIV status.

Of the 513 IDUs seropositive for anti-HCV, 414 (80.7%) had detectable HCV RNA. No anti-HCV-seronegative subjects had detectable HCV RNA, regardless the status of HIV infection. We analyzed the factors related to HCV viremia among IDUs with HCV exposure (Table 3). Age was similar between anti-HCV-seropositive IDUs with and without HCV viremia. HCV viremic subjects tended to have a higher proportion of males and higher AST and ALT ratios (*p* = 0.055, 0.08, and 0.09, respectively). Among IDUs with exposure to HCV infection, HBsAg-seropositive subjects had a significantly lower rate of HCV viremia than HBsAg-seronegative subjects did (45/78, 67.7% vs. 369/465, 79.4%, *p*<0.001). However, the status of HCV RNA did not differ between IDUs with and without anti-HIV seropositivity. Among IDUs seropositive for both anti-HCV and anti-HIV, HCV viremic subjects tended to have higher levels of HIV RNA than those without HCV viremia (*p* = 0.09). The HCV RNA status did not correlate to the CD4 cell counts. Multivariate analysis showed that among IDUs seropositive for anti-HCV, HBsAg seropositivity was strongly negatively associated with HCV viremia, whereas anti-HIV seropositivity was significantly correlated with HCV viremia (Table 4).

HCV RNA viral load was unrelated to CD4 cell counts among IDUs who were seropositive for both of HCV RNA and anti-HIV (5.26±1.25, 5.26±1.25, and 5.26±1.25 log IU/mL for IDUs with CD4 cell count >500, 350–500, and <350 cells/µL, respectively, *p* = 0.99). However, there was a significant linear correlation between HCV and HIV RNA viral loads (*R* ^2^ = 0.014, *p*<0.001).

### Molecular epidemiology of HCV infection among IDUs

The HCV genotype distribution demonstrated that genotype 6 (41.0%, 80/195) was the most prevalent genotype among IDUs, followed by genotypes 1 (32.2%, 63/195), 3 (12.8%, 25/195), and 2 (5.6%, 11/195). Subgenotype 6a/6b (36.4%) was the most prevalent subgenotype among IDUs, followed by subgenotypes 1a (18.5%) and 1b (13.8%). There were no differences in HCV genotype and subgenotype distributions between IDUs with and without HIV infection. In Table 5, we list the results for HCV genotypes among IDUs and the general population reported in previous studies [9], [19], as well as HCV genotype distributions among the general population in Taiwan [20], [21].

**Table 5:** HCV genotype distributions for anti-HIV (−) and anti-HIV (+) IDUs, along with previously reported genotype distributions among IDUs and the general population.

	HCV genotype distributions among IDUs, no. (%)						HCV genotype distributions among general population, no. (%)	
Genotype	Total	Anti-HIV(−)	Anti-HIV(+)	*p* a	Liu et al.^b^ [9]	Lee et al.^b^ [19]	Lee et al. [34]	Yu et al. [20], [21]
**Total**	**195**	**50**	**145**		**243**	**141**	**407**	**56**
**1**	**63 (32.3)** c	**17 (34.0)** d	**46 (31.7)** d		**103 (42.4)** c	**69 (48.9)** c	**201 (49.38)** c	**25 (44.64)** c
1a	36 (18.5)^c^	8 (16.0)^d^	28 (19.3)^d^	0.33^c^	71 (29.2)	21 (14.9)	11 (2.70)	—
1b	27 (13.8)^c^	9 (18.0)^d^	18 (12.4)^d^		32 (13.2)	48 (34.0)	190 (46.68)	25 (44.64)
**2**	**11 (5.6)** c	**4 (8.0)** d	**7 (4.8)** d		**20 (8.2)** c	**12 (8.6)** c	**158 (38.83)** c	**25 (44.64)** c
2a/2c	3 (1.5)^c^	0 (0.0)^d^	3 (2.0)^d^	0.12^c^	4 (1.6)	6 (4.3)	129 (31.70)	23 (41.07)
2b	8 (4.1)^c^	4 (8.0)^d^	4 (2.8)^d^		16 (6.6)	6 (4.3)	29 (7.13)	2 (3.57)
**3**	**25 (12.8)** c	**5 (10.0)** d	**20 (13.8)** d		**52 (21.4)** c	**12 (8.6)** c	**4 (0.98)** c	—
3a	23 (11.8)^c^	4 (8.0)^d^	19 (13.1)^d^	0.27^c^	49 (20.2)	11 (7.8)	4 (0.98)	—
3b	2 (1.0)^c^	1 (2.0)^d^	1 (0.7)^d^		3 (1.2)	1 (0.7)	—	—
**6**	**80 (41.0)** c	**17 (34.0)** d	**63 (43.4)** d		**68 (28.0)** c	**48 (33.9)** c	**2 (0.49)** c	—
6a/6b	71 (36.4)^c^	16 (32.0)^d^	55 (37.9)^d^	0.43^c^	57 (23.5)	43 (30.5)	2 (0.49)^e^	—
6c-1	9 (4.6)^c^	1 (2.0)^d^	8 (5.5)^d^		4 (1.6)	—	—	—
6k	—	—	—		7 (2.9)	—	—	—
6n	—	—	—		—	4 (2.8)	—	—
6w	—	—	—		—	1 (0.7)	—	—
**Mixed**	**15 (7.7)** c	**7 (14.0)** d	**8 (5.5)** d		—	—	**20 (4.91)**	**4 (7.14)**
**Unclassified**	**1 (0.5)** c	**0 (0.0)** d	**1 (0.7)** d		—	—	**22 (5.41)**	**2 (3.57)**

HCV, hepatitis C virus; HIV, human immunodeficiency virus.

a *p* for subgenotype of hepatitis C virus.

b IDUs; does not include IDUs infected with more than two HCV genotypes.

c Percentage for all cases of hepatitis C virus.

d Percentage for groups (anti-HIV(+) and anti-HIV(−)) of hepatitis C virus.

e Genotype 6a in this study.

## Discussion

HCV and HIV have similar modes of transmission. Exposure to contaminated blood or blood products, particularly via injection drug use, is the most efficient route of HCV transmission. Among injection drug users in Taiwan in the late 1990s, the prevalence of anti-HCV antibody positivity was 66.4–67.2% [22], [23]. Despite the implementation of national policies aimed at stopping the spread of HCV infection in Taiwan [24], [25], the prevalence of anti-HCV positivity in IDUs with HIV infection in Taiwan was around 97% in the late 2000s [8], [9]. In the current study, we confirmed that the prevalence of anti-HCV seropositivity was not only extremely high among HIV-infected IDUs (98.7%), but also very high among anti-HIV-negative IDUs (83%). HIV infection is a strong risk factor for HCV co-infection, with an OR of 23.642 (95% CI: 8.142–68.853), indicating that more efforts are needed to prevent the transmission of HCV among IDUs.

Taiwan is an HBV-hyperendemic area, with a prevalence rate of around 15% in the general population [17], [26] as well as in end-stage renal disease patients under maintenance hemodialysis [16]. Although the prevalence of HBsAg-seropositive IDUs in the current study was also around 15%, we found that HIV infection was associated with HBsAg seropositivity, with an OR of 1.90 (95% CI: 1.11–3.34). Similar findings were also reported among men who have sex with men [27]. These findings suggest that immunosuppressed status, such as HIV infection, might play a role in persistent HBV infection, even in areas where vertical transmission is the major route of HBV infection, such as Taiwan.

Several factors have been associated with persistent HCV viremia after acute HCV infection in the general population and end-stage renal disease patients, including age at infection, symptoms at presentation, mode of infection, rapid HCV viral decline, concomitant HBV infection, and host interleukin-28B genetic variants [15], [16], [28]–[31]. We previously demonstrated that the rate of HCV viremia after HCV exposure was around 70–75% in the general population and among end-stage renal disease patients under regular maintenance hemodialysis [16], [26], [32]. In the current study, we also found that HBV infection had interactive and suppressive effects on HCV viremia in IDUs with HCV exposure: 57.7% HBsAg-seropositive versus 84.8% HBsAg-seronegative subjects. These rates are similar to those observed among the general population (55% vs. 77%, respectively) [26] and end-stage renal disease patients (60% vs. 77%) [16]. In contrast, a 6-fold greater chance of persistent HCV viremia was noted in our anti-HCV-seropositive IDUs with HIV co-infection compared with those without HIV co-infection. These results suggested that either HIV-related immunocompromised status or HIV–HCV interaction might promote persistent HCV viremia in HIV-infected IDUs with HCV exposure. Recent reports indicated that host genetic interleukin-28B genotypes are strongly correlated with spontaneous HCV clearance in HCV-mono-infected subjects [31] or HCV/HIV co-infected patients [33]. However, there were no host genetic data available in the current study.

In the general population of Taiwan, the most prevalent HCV genotype is genotype 1b (50–60%), followed by genotype 2a (30–35%) [21], [34], [35]. Genotypes 1a, 3, and 6 infections are very rare in the general population of Taiwan. However, the molecular epidemiology of HCV is quite different between the general population and IDUs. Similar to previous studies [9], [19], [36], we found that genotype 6 (41.0%) was the most prevalent genotype among our IDUs, followed by genotypes 1a, 1b, and 3a. HCV subgenotypes 1a, 3a, and 6a are widely distributed in Southeast Asia [9], [37], and these may spread through southern China to Taiwan via the same routes used for smuggling illegal drugs that are responsible for the spread of HIV infection.

In this study, the prevalence of HCV/HIV co-infection was 52.1% (293/562), which is substantially higher than the prevalence reported by Hung et al. in Taiwan during the period from 1994 to 2002 (9.6%) [38]. This discrepancy might be due to the difference in study populations: a prison-based population in the current study versus a hospital-based population in Hung's study. The high prevalence of co-infection that we observed indicates that injection drug use is an important risk factor for HCV/HIV co-infection in Taiwan. Therefore, all HIV-infected patients should be tested for HCV infection [39]–[41]. Interestingly, we found that the anti-HCV prevalence was significantly higher in female than in male IDUs. Similar results were also observed in the Taiwanese general population (7.6% for women and 5.5% for men; OR, 1.95; 95% CI, 1.65–2.31) [26]. Why females are at higher risk of HCV exposure in both the general population and among IDUs in southern Taiwan remains to be clarified. The significantly higher rate of anti-HCV seropositivity among female subjects might be due to the gynecological intervention, abortion, ear piercing, cosmetic surgery and eyebrows tattooing. All of these behaviors are at risk of HCV exposure. However, the actual causal etiology remains to be studied.

Consistent with a previous study that HBV co-infected IDUs had a 6-fold greater chance of HCV seroclearance when compared to those who were never exposed to HBV [42], we found a reciprocal interaction between HBV infection and HCV viremia among IDUs with HCV exposure. Similar findings were also reported in the general population [15] and uremic patients under maintenance hemodialysis [43]. Further studies are needed to explore the pathophysiology of the virus–virus interaction between the two hepatotropic viruses in the same host [44], [45].

One limitation of the current study is that we did not collect data on the duration and nature of IDUs behavior, which might have a great impact on the risk for HCV and HIV acquisition. The Center for Disease Control, Taiwan, has conducted a series of harm-reduction programs, including health education, providing clean syringes, and methadone substitution treatment, to reduce the risk of HIV and viral hepatitis transmission among IDUs (http://www.cdc.gov.tw/english/page.aspx?treeid=e79c7a9e1e9b1cdf&nowtreeid=6bb9113c9e323e98). However, based on the results of the current study, there is still room to improve the efficacy of these strategies in Taiwan.

## Conclusions

Among Taiwanese IDUs, more than half were infected with HIV and more than 90% were infected with HCV. Male gender, HBsAg seropositivity, and anti-HCV exposure were factors related to HIV infection among IDUs. Those with HIV infection had a 6-fold greater chance of developing persistent HCV viremia, whereas subjects with HBV infection had a 6-fold greater chance of spontaneous HCV seroclearance. Our results also highlight the different HCV molecular epidemiology among IDUs in Taiwan. Our findings could provide helpful information for management and decision-making in the clinical settings.

## Supporting Information

Figure S1 The prevalence of HIV infection were 0% (0/5), 9.1% (4/44), 27.6% (43/156) and 70% (250/357), respectively, for anti-HCV-seronegative female patients, anti-HCV-seronegative male patients, anti-HCV-seropositive female patients and anti-HCV-seropositive male patients (*p* for trend <0.001, Figure S1).
(TIF)
