# Supplementary figures and images for: Hepatitis C Virus Infection among Injection Drug Users with and without Human Immunodeficiency Virus Co-Infection
Source: PLoS One. 2014 Apr 10;9(4):e94791. doi: 10.1371/journal.pone.0094791 (PMC3983255; doi:10.1371/journal.pone.0094791)

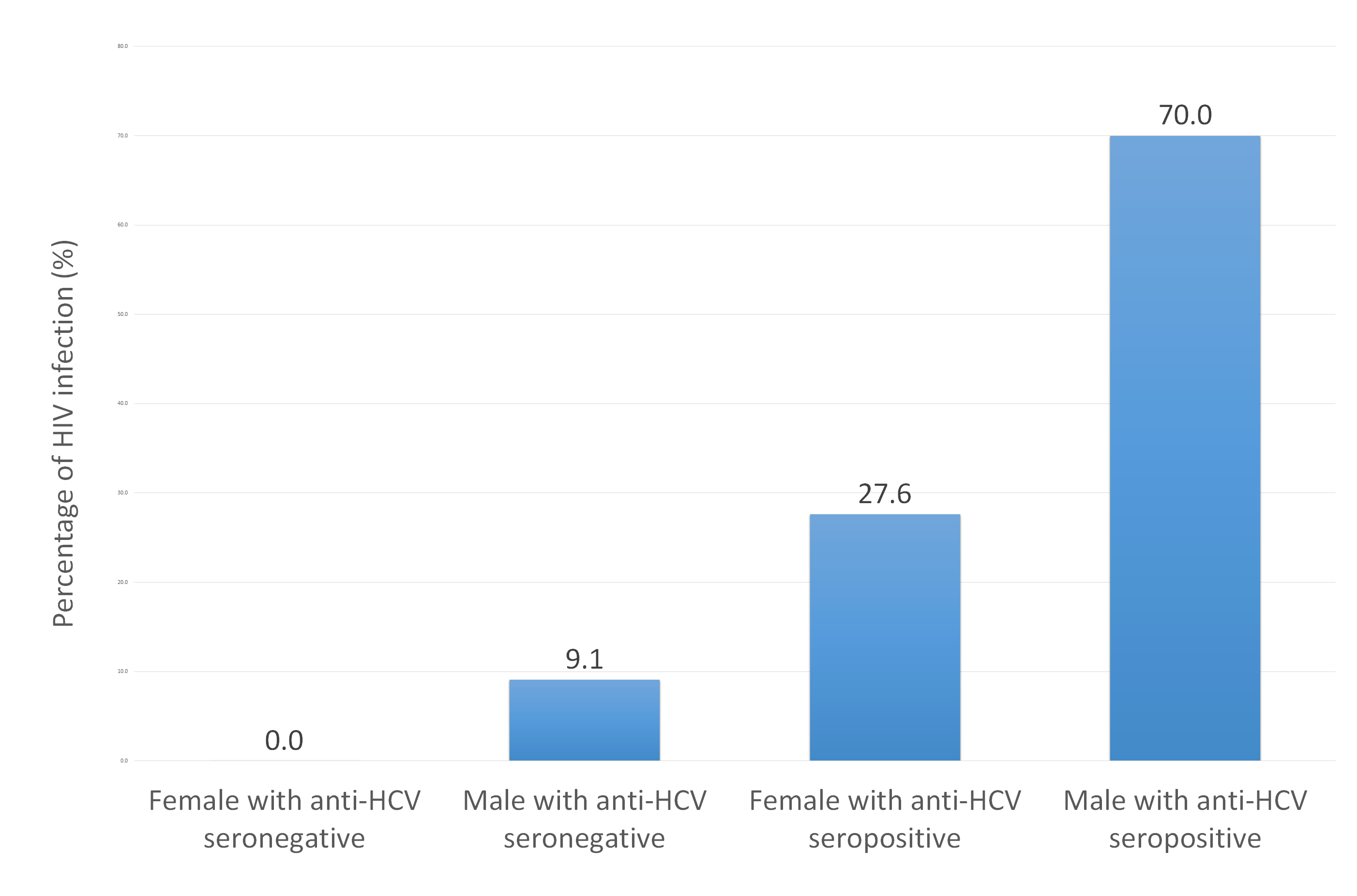

Supplement: Figure S1 — The prevalence of HIV infection were 0% (0/5), 9.1% (4/44), 27.6% (43/156) and 70% (250/357), respectively, for anti-HCV-seronegative female patients, anti-HCV-seronegative male patients, anti-HCV-seropositive female patients and anti-HCV-seropositive male patients (p for trend <0.001, Figure S1). (TIF) [file pone.0094791.s001.tif]
